# Depth Profilometry via Multiplexed Optical High-Coherence Interferometry

**DOI:** 10.1371/journal.pone.0121066

**Published:** 2015-03-24

**Authors:** Farnoud Kazemzadeh, Alexander Wong, Bradford B. Behr, Arsen R. Hajian

**Affiliations:** 1 Department of Systems Design Engineering, University of Waterloo, Ontario, Canada; 2 Tornado Spectral Systems, Toronto, Ontario, Canada; Washington State University, UNITED STATES

## Abstract

Depth Profilometry involves the measurement of the depth profile of objects, and has significant potential for various industrial applications that benefit from non-destructive sub-surface profiling such as defect detection, corrosion assessment, and dental assessment to name a few. In this study, we investigate the feasibility of depth profilometry using an Multiplexed Optical High-coherence Interferometry MOHI instrument. The MOHI instrument utilizes the spatial coherence of a laser and the interferometric properties of light to probe the reflectivity as a function of depth of a sample. The axial and lateral resolutions, as well as imaging depth, are decoupled in the MOHI instrument. The MOHI instrument is capable of multiplexing interferometric measurements into 480 one-dimensional interferograms at a location on the sample and is built with axial and lateral resolutions of 40 μm at a maximum imaging depth of 700 μm. Preliminary results, where a piece of sand-blasted aluminum, an NBK7 glass piece, and an optical phantom were successfully probed using the MOHI instrument to produce depth profiles, demonstrate the feasibility of such an instrument for performing depth profilometry.

## Introduction

Depth profilometry is the measurement of the depth profile of objects, and has seen widespread interest for various industrial applications such as defect detection [1, 2], corrosion assessment, and dental diagnosis [3–5]. A number of instruments have been proposed for performing depth profilometry, ranging from photothermal radiometry [1, 4, 5] and photocarrier radiometry [2] to luminescence [4, 5] and photo-thermo-acoustic [6]. In this study, we investigate the feasibility of depth profilometry from an optical interferometry perspective via an Multiplexed Optical High-coherence Interferometry (MOHI) instrument.

Here, the MOHI instrument utilizes a laser source to acquire multiple interferograms simultaneously at the same location, which are then combined to obtain the reflectivity as a function of depth to measure the depth profile of the sample. Extending significantly beyond the basic principles first conceptualized in [7], the MOHI instrument introduced for this study capitalizes on the spatial coherence of a laser and the interferometric properties of light to probe into a sample to measure its depth profile using a multiplexed interferometry strategy. MOHI is a wavefront-splitting interferometer, popular in particle interferometry [8, 9], since it samples two disjoint sections of the scattered light beam returning from a sample and, by overlapping the two, produces an interference pattern. From the interference pattern a depth profile can be reconstructed. In MOHI the lateral resolution, the axial resolution, and the maximum imaging depth are independent of each other. The lateral resolution is determined by the probing spot size, the axial resolution is dependent on various design choices of the system, and the imaging depth limitation is directly proportional to the coherence length and power of the laser utilized. In this case, a laser diode with coherence length of 700 *μ*m corresponds to the maximum reliable imaging depth measured in MOHI. However, using frequency-stabilized lasers, coherence lengths on the order of millimeters can be achieved, hence dramatically increasing the imaging depth limit [10].

The MOHI instrument is designed based on the *a priori* assumption that a depth profile can be retrieved from an interference pattern. The overlap of the light beams from the two sub-apertures, sampled from the full collimated aperture of backscattered light, would give rise to this interference pattern. The interference pattern is observed on the sensor plane of the detector if the two beams are spatially coherent.

## Materials and Methods

**Fig 1:**
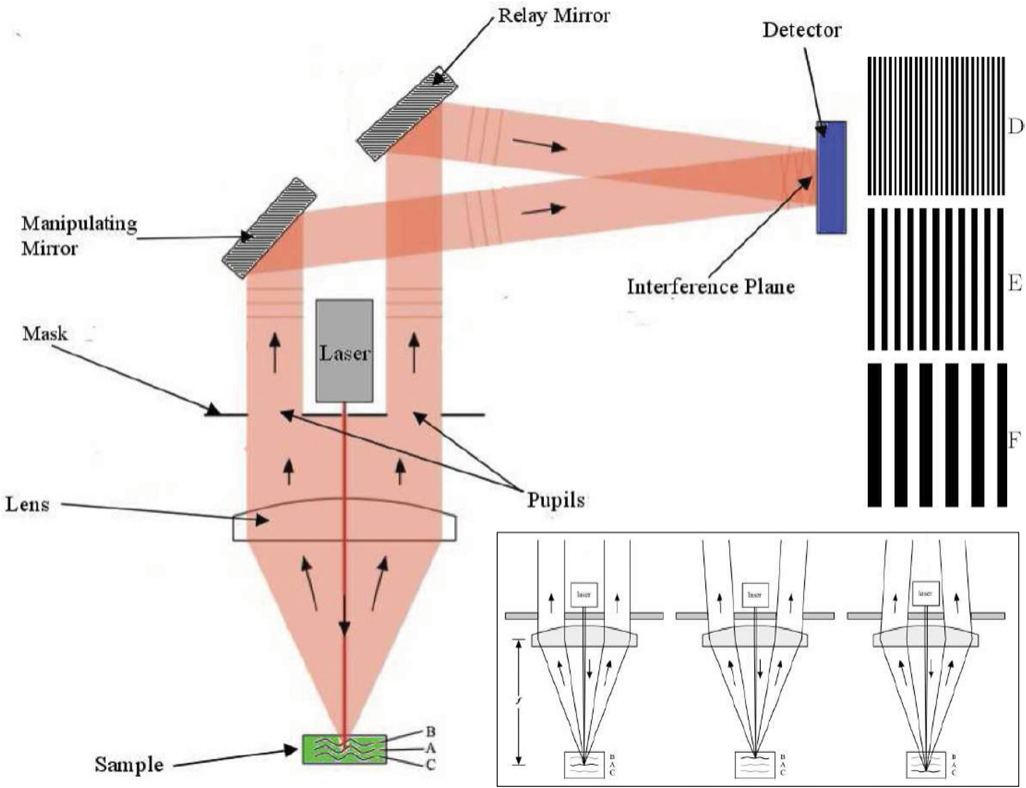
The schematic of the optical design of MOHI for depth profilometry. If a scattering surface is placed at the focal plane of the instrument the two beams emerging from the sub-apertures are parallel, otherwise the two beams will diverge or converge as shown in the inset figure. Using the manipulating mirror, an artificial OPD is created which result in a two-dimensional interference pattern of a prescribed frequency. As different depths of a sample are probed the OPD will be altered which would result in different peaks in the frequency spectrum signifying the return of the signal from a different depth other than the focal plane. Note that the notion of ‘depth’ is conceived to be a deviation from the focal plane of the instrument and it can be myopic or hyperopic. This is graphically demonstrated using the three different surfaces (B, A, and C) in the sample with their correspondent two-dimensional interferograms D, E, and F observed on the detector.

The schematic of the optical design of MOHI is shown in Fig. 1. The light from the laser source is focused on the surface of the sample by a lens. The light is then scattered off of the surface or from a depth within the sample in all directions in the hemisphere toward the lens. The same lens collimates the backscattered light on its way to a mask, which only allows the light that pass through two sub-apertures to continue down-stream. The light beams emerging from the sub-apertures are then steered by two mirrors: i) a relay mirror which bends the path of the beam and places it onto the detector, and ii) a manipulating mirror that overlaps the beams at the imaging plane of the detector. The process of overlapping the two beams introduces an optical path difference (OPD) between the two beams. The beams emerging from the sub-apertures can be expressed as an intensity profile described by,

$$\begin{array}{c} I=\epsilon \;\mu <ℛ\{E^{2}\}>, \end{array}$$ (1)

where *ε* and *μ* are the permitivity and the permeability of the medium that the waves are traveling in, air in this case, and < ℛ{*E* ^2^} > is the time average of the square of the real part of the electromagnetic radiation. The observed interference is the results of an OPD, Δ*ϕ*, described by,

$$\begin{array}{c} I=\epsilon \;\mu \;[I_{0}+I_{1}+2\sqrt{I_{o}I_{1}}\;cos\left(\Delta \varphi \right)], \end{array}$$ (2)

with *I*_0_ and *I*_1_ being the intensities of each one of the sub-aperture beams.

The OPD between the two beams emerging from the sub-apertures changes depending on the location of a scatterer or a scattering event with respect to the focal plane of the instrument. The varying OPD between the two sub-aperture beams result in a two-dimensional interference pattern that is captured by a two-dimensional detector. Each row of the two-dimensional interferogram captured by the detector corresponds to an individual one-dimensional interferogram acquisition. As such, the MOHI instrument effectively acquires multiple one-dimensional interferograms simultaneously at the same location on the sample. The spatial frequencies of the interferograms correlate with the depth at which the light is scattering from. The intensity of the corresponding power spectrum will correspond to the backscattering power at each depth capitulating to the decrease of the instrumental signal-to-noise ratio (SNR).

In general, referring to Fig. 1, if the scattering surface is encountered at the focal plane of the instrument the beams emerging from the two sub-apertures in the mask will be parallel *à la* surface ‘A’ in Fig. 1. If the scattering surface is in front or behind the focal plane of the instrument, the beams emerging from the sub-apertures will no longer be collimated and will be under- or over-collimated, respectively, *à la* surfaces ‘B’ and ‘C’. Based on this, the sub-aperture beams will have three states: i) parallel, ii) diverging, and iii) converging. The curvature of the under- and over-collimated wavefront can be neglected and approximated to be flat in the far field especially when sampled over a small area [11].

The frequency of the interference pattern observed on the detector can be written as

$$\begin{array}{c} ℱ=\frac{d}{L\;\lambda }\;\left[1+\frac{4z\;l_{mm}}{d^{2}+4f^{2}}\right], \end{array}$$ (3)

where *d* is the separation distance between the sub-apertures on the mask, *L* is the distance between the relay mirror and the detector, *λ* is the wavelength of the light source, *l*_*mm*_ is the distance between the mask and the relay mirror, *f* is the focal length of the lens, and *z* is the distance of a scattering event from the focal plane of the instrument [12]. Note that the distance, *z*, could be positive or negative depending on the location of the backscattering surface with respect to the focal plane. There is a clear connection with the frequency of the interference pattern recorded by the detector and the distance at which there exists a backscattering event.

### Optical Design

The light source used in the MOHI instrument is a 5 mW, 653 nm solid-state laser diode powered by a stabilized 3 V DC power supply. The light is focused onto the sample by a plano-convex singlet objective lens with numerical aperture of 0.22. The same lens is employed to collect the scattered light returning from the sample. Simulation and experimental results have shown that angular distribution of backscattered light after interacting with a sample is anisotropic above its surface [13]. The light backscatter distribution tends to be Gaussian about the optical axis with the $\frac{1}{e^{2}}$ (13% of the peak) waist at ∼ 13° [13, 14]. As a result, an f/2 lens (NA = 0.22) is selected. To accommodate the size of the relay and manipulating mirrors along with their mounting hardware as well as allowing for the largest separation of the two sub-apertures in the mask within the clear aperture, a 50 mm diameter lens was used. Another consideration for selecting an appropriate lens is to select a very fast lens (high NA). Such a lens will improve the lateral imaging resolution and decrease the depth of field of the instrument which will result in higher resolution in the OPD. The mask is a piece of black anodized aluminium which acts as a beam dump, with the exception of the two sub-apertures to minimize stray light in the device. The size of and the separation between the sub-apertures, 5 mm diameter and 37 mm respectively, are optimized for throughput given the current configuration of the instrument while maximizing the axial resolution and the accessible imaging depth of the instrument. The relay mirror and the manipulating mirror in MOHI are broadband dielectric mirrors that offer > 99% reflectivity at the desired wavelength (653 nm). The manipulating mirror is mounted on a kinematic mount for ease of control over tip and tilt of the beam reflecting off of it.

The two-dimensional interference pattern is recorded using an 8-bit monochrome CMOS detector with a chip size of 4.5 mm × 2.9 mm and a 6 *μ*m pixel pitch. The properties of the detector place limitations and restrictions on other design aspects as well as the performance and capabilities of the instrument. The MOHI detector does not perform any imaging since the detector does not use an imaging lens. The two beams of the instrument are simply overlapped on the detector sensor plane to create the interferogram.

Adhering to the Nyquist-Shannon theorem [15, 16], the highest possible fringe frequency observed using a detector is when one fringe is observed across two pixels, meaning that one detector pixel is attributed to constructive interference and one to the destructive interference. Depending on the pixel size of a detector, this maximum fringe frequency will be different which will in turn affect the axial resolution and the overall size of the instrument as demonstrated in Equation 3 and Equation 5. Additionally, the size of the detector chip and its bit depth plays an important role in determining the maximum imaging depth of the instrument.

The sample being measured by MOHI is placed on a platform which allows for X-Y-Z displacement. This allows for focus control of the instrument by moving the sample toward or away from the lens and translation in the other two dimensions enables two- and three-dimensional sensing. The distance between the mask and the relay mirror and between the relay mirror and the detector is 448 mm and 892 mm, respectively.

The axial resolution of the MOHI instrument can be determined by considering the smallest deviation from the focal plane of the instrument that would result in an addition of or subtraction of one fringe from the interferogram. As such, Equation 3 can be written as,

$$\begin{array}{c} N_{F}=\frac{d\;N_{px}\;\delta_{px}}{L\;\lambda }\;\left[1+\frac{4z_{\circ }\;l_{mm}}{d^{2}+4f^{2}}\right],\;and,\;\left(N_{F}+1\right)=\frac{d\;N_{px}\;\delta_{px}}{L\;\lambda }\;\left[1+\frac{4z_{1}\;l_{mm}}{d^{2}+4f^{2}}\right], \end{array}$$ (4)

where *N*_*F*_ is the number of fringes observed on the detector at depth *z*_∘_, (*N*_*F*_+1) is the number of fringes observed at depth *z*_1_, *N*_*px*_ is the number of detector pixels and *δ*_*px*_ is the size of each pixels. Rearranging the equations above and letting Δ*z* = *z*_1_ − *z*_∘_, the smallest axial distance discernible is

$$\begin{array}{c} \Delta z=\frac{L\;\lambda }{l_{mm}\;\left(N_{px}\;\delta_{px}\right)}\;\left(\frac{d}{4}+\frac{f^{2}}{d}\right), \end{array}$$ (5)

As previously discussed the size of the detector pixel plays a part in determining the axial resolution of MOHI. Based on the current prototype, the theoretical axial resolution is 40 *μ*m. This was experimentally verified by displacing the aluminium block at 20 *μ*m steps in front and behind the focal plane and capturing the corresponding depth profile. The depth profile measured at 0 *μ*m, ± 40 *μ*m, and ± 80 *μ*m were observed at the expected depth but the depth profiles measured at ± 20 *μ*m and ± 60 *μ*m were observed to be at 40 *μ*m and ± 80 *μ*m. This demonstrates that the smallest discernible feature in the axial dimension is 40 *μ*m. The lateral resolution is determined by simply using the Rayleigh criterion [17], and is determined to be approximately 40 *μ*m.

### Interferogram Multiplexing

**Fig 2:**
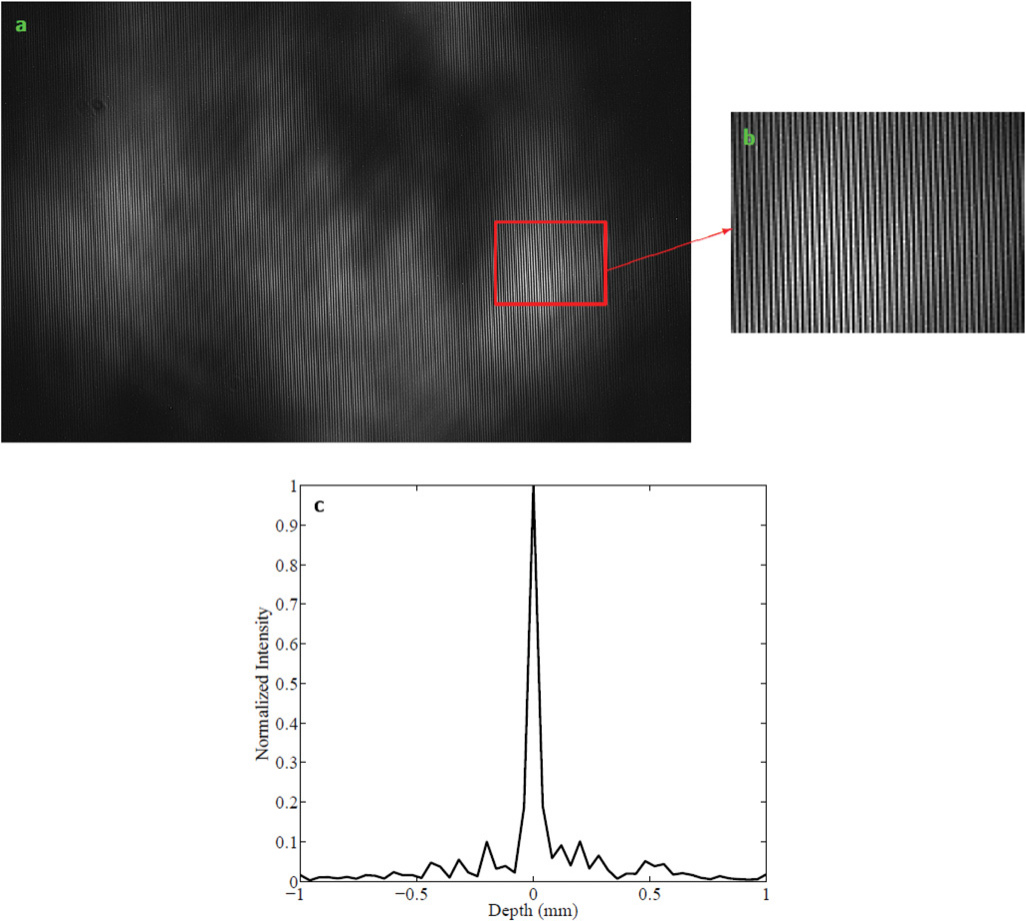
Point spread function of MOHI. a) The interferogram observed and recorded by the detector with a specific region of interest bordered by the red rectangle. b) A zoom-in of the region of interest. c) The PSF of MOHI produced by performing a Fourier transform on the recorded interferogram.

A depth profile is constructed by calculating the frequency of the interference pattern, usually using a Fourier transform. In the MOHI instrument, the acquired two-dimensional interferogram, shown in Fig. 2a, is multiplexed into many one-dimensional interferograms which are then used to create independent depth profiles. The current configuration of the instrument is capable of multiplexing the two-dimensional interferogram into 480 one-dimensional interferograms which is directly proportional to the number of rows of pixels on the detector. Due to fluctuations rising from photon statistics and behavior of lasers in general, only the interferograms, from the set of 480, with visibility larger than 75% are used for processing and reconstruction of the depth profile. The visibility of an interferogram is determined by

$$\begin{array}{c} {𝒱}_{j}=\frac{I_{j}^{max}-I_{j}^{min}}{I_{j}^{max}+I_{j}^{min}}, \end{array}$$ (6)

where *I* ^*max*^ and *I* ^*min*^ are the maximum and minimum intensity of the interference pattern, and is one form of determining the signal-noise relationship of an interferogram [17]. The subscript *j* is being used to denote each one-dimensional interferogram of the two-dimensional interferogram recorded. On average, ∼ 60% of the 480 interferograms had a visibility greater than the threshold. Generally, the depth profiles produced by the individual one-dimensional interferograms have low signal-to-noise ratio (SNR). To address the low SNR issue, the depth profiles produced by multiplexed one-dimensional interferograms are then combined based on the principle of Bayesian Maximum A Posteriori (MAP) inferencing [18–21] to produce a more reliable depth profile with increased SNR.

### High SNR Depth Profile Reconstruction

An important challenge faced in reconstructing a depth profile from a single one-dimensional interferogram is the issue of SNR. Given that the proposed MOHI instrument produces a set of one-dimensional interferograms, a simple approach to improving SNR is to simply average the reconstructed depth profiles obtained from each interferogram together. However, such an approach does not account for the intrinsic instrument properties and as such is limited in its ability to produce a high quality depth profile reconstruction. Motivated by this, we propose to further improve the SNR of the depth profile by accounting for the intrinsic instrument properties within a Bayesian MAP inferencing framework.

A one-dimensional interferogram can be modeled as a linear shift-invariant phenomenon and can be described by $\hat{g}=\hat{f}\;*\;h$, where $\hat{g}$ is the Fourier transform of the observed interferogram *g*, $\hat{f}$ is the expected depth profile, *h* is the point spread function (PSF) of the instrument, shown in Fig. 2c, which is the Fourier transform of the optical transfer function, and * denotes convolution of the two functions. The concept of Bayesian MAP inferencing can be used to reconstruct a depth profile, $\hat{f}$, given *h* and $\hat{g}$, as follows:

$$\begin{array}{c} \hat{f}=argmax_{\hat{f}}\;P\left(\hat{f}|\hat{g},h\right). \end{array}$$ (7)

The *argmax* function is a mathematical term that stands for argument of the maximum and is used to describe the desire to maximize the objective function, which in our case is to find the theoretical depth profile $\hat{f}$ that maximizes the conditional probability of $\hat{f}$, given the measured depth profile, $\hat{g}$, and the instrument PSF, *h*.

Here, if we were to treat $\hat{g}$ and $\hat{f}$ as Poisson statistical phenomena, we can arrive at the following Poisson MAP formulation for the iterative depth profile reconstruction:

$$\begin{array}{c} {\hat{f}}_{n}={\hat{f}}_{n-1}\;exp\left[\left(\frac{\hat{g}}{{\hat{f}}_{n}*h}-1\right)*h^{+}\right], \end{array}$$ (8)

where ${\hat{f}}_{n}$ is the reconstructed depth profile at iteration *n* based on the most recent estimate ${\hat{f}}_{n-1}$ at iteration *n* − 1, and *h* ^+^ is the adjoint of *h*. The *n* subscript is used to denote that the reconstruction of the depth profile is an iterative process which produces a new depth profile estimate based on the previous calculated estimate. At iteration *n* = 2, the most recent estimate ${\hat{f}}_{1}$ is simply the measured depth profile without any SNR improvement.

The MAP reconstruction algorithm is designed to iteratively improve the SNR of the depth profile. The iterative process is repeated until there is little marginal SNR improvement of the current depth profile compared to the previous depth profile.

Given the fact that a collection of one-dimensional interferograms (denoted by *g*_1_, …, *g*_*N*_) is available as a result of the aforementioned multiplexing process, one can extend the aforementioned Bayesian MAP formulation to take advantage of all of the one-dimensional interferograms available to achieve a high-fidelity reconstruction of the depth profile, Equation 7 can be written as

$$\begin{array}{c} \hat{f}=argmax_{\hat{f}}\;P\left(\hat{f}|{\hat{g}}_{1},\cdots ,{\hat{g}}_{N},h\right), \end{array}$$ (9)

where ${\hat{g}}_{i}$ is the Fourier transform of the *i* ^*th*^ observed interferogram. Finally, the Poisson MAP iterative optimization formulation, Equation 8, is given by,

$$\begin{array}{c} {\hat{f}}_{n}={\hat{f}}_{n-1}\;exp\left\{\frac{1}{N}\left[\left(\frac{{\hat{g}}_{1}}{{\hat{f}}_{n}*h}-1\right)*h^{+}\right]\right\}\cdots exp\left\{\frac{1}{N}\left[\left(\frac{{\hat{g}}_{N}}{{\hat{f}}_{n}*h}-1\right)*h^{+}\right]\right\}. \end{array}$$ (10)

This formulation now considers all multiplexed one-dimensional interferograms in product in the iterative reconstruction process. Therefore, the MAP reconstruction method used to reconstruct a depth profile from the set of one-dimensional interferograms can be summarized in three steps: 1) set initial solution ${\hat{f}}_{1}$ as the measured depth profile without SNR improvement, 2) perform Equation 10 until there is little marginal SNR improvement in the reconstructed depth profile, and 3) the final reconstructed depth profile is set to ${\hat{f}}_{m}$, where *m* is the termination iteration. Based on empirical tests in this study, termination iteration where there is little marginal SNR improvement is reached when *m* is ∼ 150.

### Sample Preparation

A sample which can produce specular scattering with no depth information is deemed to be ideal for calibration and characterization of the MOHI instrument. A piece of sand-blasted aluminum was used for this purpose. To evaluate the feasibility of depth profilometry using MOHI, an NBK7 glass piece was shaved down to a thickness of 360 *μ*m and both outer surfaces were sanded to create more pronounced air-glass transition boundaries. For further feasibility analysis, a Biomimic Optical Phantom was acquired from the National Optics Institute, Quebec, Canada [22]. This phantom is a solid block of size 2 × 2 × 1 cm (L × W ×H) and was designed to possess optical properties similar to human breast tissue. This phantom was machined using a precision CNC milling machine to have a staircase pattern of four platforms 5 mm long with transition height of 250 *μ*m between each step.

## Results

**Fig 3:**
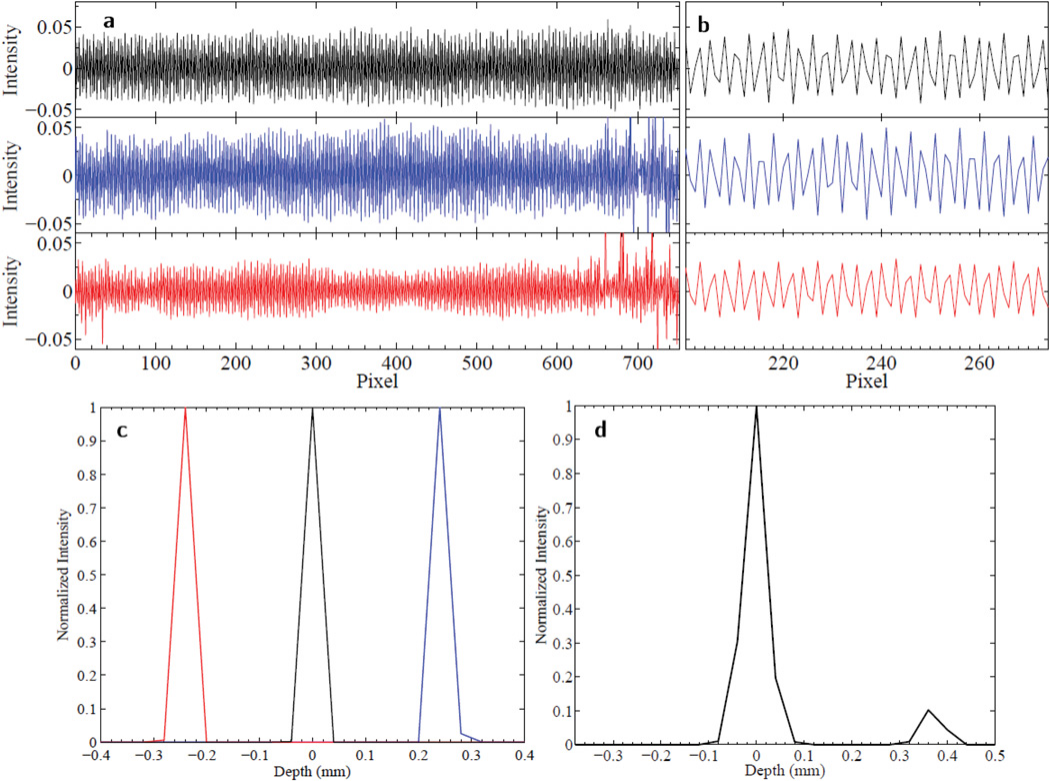
Experimental results for one-dimensional depth profilometry using MOHI. a) Sinusoidal interferograms observed by MOHI by placing an aluminum block at different distances from the lens, at the focal plane (black) and 240 *μ*m in front and behind the focal plane (red and blue respectively). b) A closer look of the interferograms shown in (a). c) The depth profiles of the aluminum block at three different depths, note that positive depth and negative depth indicate the observation plane is behind and in front of the focal plane of the instrument, respectively. d) The depth profile of a 360 *μ*m thick glass sample measured with MOHI.

A prototype MOHI instrument was built and a number of experiments were performed to assess the feasibility of performing depth profilometry using MOHI, as well as to verify the theoretical principles on which MOHI is based on. In the first experiment, an aluminum block was probed at three different axial locations with respect to the focal plane of the instrument. Fig. 3a shows one of 480 corresponding one-dimensional interferograms and Fig. 3b shows a portion of the same interferograms. It can be observed that the one-dimensional interferograms can be approximated by a zero-mean sine function, also that the number of peaks observed in the same range of the data (Fig. 3b) is different depending on where the block was placed with respect to the focal plane. Fig. 3c shows the depth profiles reconstructed from the interferograms. Of note is the fact that interferogram SNR is highest when the sample is placed at the focal plane of the instrument and decreases away from focal plane, however, the SNR of the depth profile is unchanged over the imaging depth limit using the reconstruction method previously described. The depth profiles accurately indicate the location of the surface of the block at ± 240 *μ*m. The axial resolution of the instrument, which is determined by the half-Rayleigh criterion, is calculated to be 40 *μ*m. The full-width-half-maximum of the PSF shown in Fig. 2c correspond to the theoretical value.

In the second experiment, the feasibility of performing depth profilometry using MOHI was investigated using a glass sample with two highly scattering surfaces separated by a known thickness. Fig. 3d shows the depth profile of the 360 *μ*m thick glass sample measured using MOHI. The MOHI instrument was able to detect both the front and the back surface of the glass sample. The back surface of the glass sample appears to have a much lower rate of scattering which can be attributed to the lower SNR as a function of depth as well as the fact that the sample was suspended in air while making the measurement. The transition of index of refraction from glass to air results in a lower backscatter than the reverse. The separation of 360 *μ*m between the front and the back surface of the glass sample is accurately measured by the MOHI instrument within the 40 *μ*m uncertainty in the axial resolution of the instrument.

**Fig 4:**
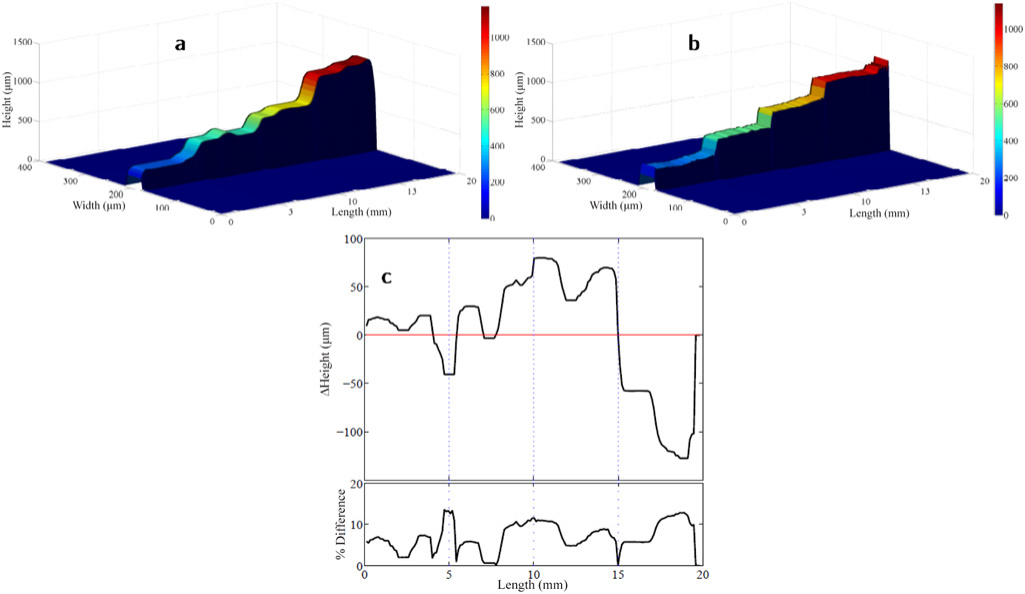
Profile of the staircase pattern of the biomimic optical phantom. a) measured by MOHI and b) measured by OCT for reference. c) the difference between the profiles measured using the MOHI and the reference profile (upper) and the percent difference between the two profiles (lower), as a visual aid the line of equality is shown in red and the locations of step transitions are shown in blue.

In the third experiment, the prototype MOHI instrument was used to acquire the surface profile of a biomimic optical phantom (Fig. 4). The phantom was machined to have three height transitions with step size of 250 *μ*m each. The phantom was then observed using MOHI, Fig. 4a, which was able to discriminate between the four steps. For reference, the phantom was also observed using an optical coherence tomography (OCT) system with axial and lateral resolution of 6 *μ*m (Fig. 4b). The reference profile demonstrates that the machining process to create the four steps was not done very accurately and there exists small deviations from the expected height. The measured profile acquired using MOHI was compared with the reference profile to examine the efficacy of the profile produced by MOHI. The profile measured using MOHI is at a lower lateral resolution than the OCT profile, hence, we up-sampled the lateral resolution of MOHI to match the lateral resolution of the OCT. Fig. 4c shows the absolute and percentage difference between the measured and the reference profile. The standard deviation of the difference map is ∼ 47 *μ*m which is slightly larger than the axial resolution of MOHI at 40 *μ*m.

## Discussion

Some of the performance parameters for the MOHI instrument are presented in Table 1. The current MOHI instrument is optimized for a single scattering surface, however, depth profilometry of two scattering surfaces separated in the axial dimension is successfully demonstrated using the glass sample, as previously discussed. A full mathematical treatment of the wave interference would suggest that multiple scattering surfaces would result in interference cross-terms in Equation 2. This would raise a fundamental issue concerning the operating principle of MOHI, namely the mixing of *m* ^2^ − *m* interference cross-terms (*m* is the number of scattering surfaces or changes in the index of refraction) into the observed interferogram. This effect was not fully investigated and is beyond the scope of the study reported here, although it will be considered a topic for further investigation. It can be posited that the effects of the interferometric cross-terms can be overcome by employing the multiplexing approach where interferograms with poor visibility are omitted from the reconstruction process, or by employing the MAP reconstruction algorithm to suppress their effect. In the glass sample experiment, the expectation is to observe a total of four frequencies in the interferogram, two resulting from the cross-terms, which in turn would result in four peaks in the depth profile, however, with reference to Fig. 4d, this is not the case. This evidence seems to support the claim made above about the suppression of the cross-term by multiplexing and MAP reconstruction, however further investigation is required to fully justify this claim.

**Table 1:** The performance parameters and characteristics for MOHI.

**Parameter**	**Value**
Axial resolution (n = 1.0)	40 *μ*m
Lateral resolution (n = 1.0)	40 *μ*m
Max. imaging depth	700 *μ*m
Typical multiplexing exp. time	30 ms

According to Fig. 1 if a scatterer is located at the focal plane of the lens the two beams emerging from the sub-aperture will be parallel (collimated). If the scatterer is located farther from the lens (deeper within the sample) the beams emerging from the sub-apertures will be converging (over-collimated). Conversely, if the scatterer is located closer to the lens (shallower than the focal plane) the beams emerging from the sub-apertures will be diverging (under-collimated). The angle of the emerging beams from the mask will be subject to change depending on the location of the scatterers within a sample. With the current design, the collimated laser beam from the laser source has a diameter of 3 mm. This in turn corresponds to a depth of field of more than 6 mm determined using

$$\begin{array}{c} \Delta f=2\;\frac{1.22^{2}\;\pi \;\lambda_{c}\;f^{2}}{n\;D^{2}}, \end{array}$$ (11)

where *λ*_*c*_ is the central wavelength of the light source, *D* is the size of the aperture, and *n* is the index of refraction which is assumed to be one for simplicity [17, 23]. Therefore theoretically the imaging capability of MOHI is up to 6 mm in depth however this is not attainable due to other limitations in the instrument, more specifically the coherence length of the laser source.

Koch *et al.*[24] showed that for a linear OCT, operating on the similar interferometric principles of MOHI they would need a detector with 10,000 linear pixels to accomplish an imaging depth of 2 mm since depth information is coded into a time-dependent signal. Hence, they employed a mask to downconvert the interference pattern onto a conventional detector readily available. The instrument proposed here encodes depth information into the spatial frequency of the interference fringes observed. The fringe frequency is inversely proportional to the detector’s pixel pitch. Smaller detector pixels can record signal of higher fringe frequency. The change in the fringe frequency determines the depth at which the light is backscattered from according to Equation 3. The detector used in the current version of the MOHI instrument, with 6 *μ*m pixel pitch, is limited to recording 84 lines per millimeter which corresponds to a maximum imaging depth of ∼ 7 mm, however, as mentioned previously this is not attainable with the current MOHI instrument.

Lastly, imaging systems consisting of a lens and a detector suffer from the degradation of the modulation transfer function (MTF) also known as a ‘focusing error’ [25]. The MTF describes a limit at which information higher than a certain spatial frequency can not be discerned. Most interferometric instrument suffer from the ramification of this phenomenon since they all aim to image the interferogram by focusing it onto the detector. The proposed MOHI instrument does not use a lens to focus the interferogram onto the detector thereby not being limited to the effects of MTF. The limiting factors in discerning spatial fringe frequency in MOHI is the Nyquist-Shannon limit as well as the discontinuous nature of detector pixels.

## Conclusions

In summary, the preliminary results shown here illustrate that it is feasible to perform depth profilometry using MOHI. We do acknowledge the fact that in this study we have only demonstrated the ability to measure the separation distance between two scattering surfaces, however we believe that this method can be generalized to many scattering surfaces given the availability of appropriate targets (i.e., phantoms). The future generation of this instrument will be able to achieve higher axial and lateral resolution as well as larger imaging depths. Other performance factors of MOHI based on varying the design will be investigated. For instance, addition of more sub-apertures to increase the visibility of the interferogram created by the main beams while suppressing the visibility of the interferograms created due to the cross-terms. It is also posited that the orientation of the two sub-aperture could favor higher sensitivity toward scatterers of specific shape or size within the sample being probed ergo different aperture orientation will also be investigated to explore the possible sensing bias.
